# Optimization of transfer learning based on source sample selection in Euclidean space for P300-based brain-computer interfaces

**DOI:** 10.3389/fnins.2024.1360709

**Published:** 2024-07-12

**Authors:** Sepideh Kilani, Seyedeh Nadia Aghili, Yaser Fathi, Andreea Ioana Sburlea

**Affiliations:** ^1^Department of Biomedical Engineering, School of Electrical Engineering, Iran University of Science and Technology (IUST), Tehran, Iran; ^2^Institute of Neuroscience, Universite Catholique de Louvain, Brussels, Belgium; ^3^Bernoulli Institute of Mathematics, Computer Science and Artificial Intelligence, Faculty of Science and Engineering, University of Groningen, Groningen, Netherlands

**Keywords:** P300 event-related potential, Euclidean alignment, convolutional neural network, source sample selection, discriminative restricted Boltzmann machine

## Abstract

**Introduction:**

Event-related potentials (ERPs), such as P300, are widely utilized for non-invasive monitoring of brain activity in brain-computer interfaces (BCIs) via electroencephalogram (EEG). However, the non-stationary nature of EEG signals and different data distributions among subjects create significant challenges for implementing real-time P300-based BCIs. This requires time-consuming calibration and a large number of training samples.

**Methods:**

To address these challenges, this study proposes a transfer learning-based approach that uses a convolutional neural network for high-level feature extraction, followed by Euclidean space data alignment to ensure similar distributions of extracted features. Furthermore, a source selection technique based on the Euclidean distance metric was applied to measure the distance between each source feature sample and a reference point from the target domain. The samples with the lowest distance were then chosen to increase the similarity between source and target datasets. Finally, the transferred features are applied to a discriminative restricted Boltzmann machine classifier for P300 detection.

**Results:**

The proposed method was evaluated on the state-of-the-art BCI Competition III dataset II and rapid serial visual presentation dataset. The results demonstrate that the proposed technique achieves an average accuracy of 97% for both online and offline after 15 repetitions, which is comparable to the state-of-the-art methods. Notably, the proposed approach requires <½ of the training samples needed by previous studies.

**Discussion:**

Therefore, this technique offers an efficient solution for developing ERP-based BCIs with robust performance against reduced a number of training data.

## 1 Introduction

Brain-computer interfaces (BCIs) establish a communication link that directly connects the human brain to external devices (Yadav et al., 2020; Kawala-Sterniuk et al., 2021). BCIs primarily enable individuals with neuromuscular disorders, such as amyotrophic lateral sclerosis (ALS) and spinal cord injury, to communicate with the outside world (Abiri et al., 2019; Bonci et al., 2021). There are various types of BCI-based brain signals, including magnetoencephalography (Mridha et al., 2021; Peksa and Mamchur, 2023), electrocorticography (Miller et al., 2020), functional magnetic resonance imaging (Guo, 2020), and electroencephalogram (EEG)-based systems (Rashid et al., 2020; Ydaav and Maini, 2023). EEG-based BCIs have gained significant attention owing to their non-invasiveness, affordability, high temporal resolution, and widespread availability (Rashid et al., 2020; Ydaav and Maini, 2023).

The human brain produces different types of event-related potentials (ERPs) in response to particular stimuli (McWeeny and Norton, 2020). The present study focuses on the P300-based ERP, which is a positive peak observed in the EEG waveform that is associated with cognitive information processing in the brain (Valakos et al., 2020). The amount of stimulus information is reflected by the amplitude of the P300 wave, with larger P300 waves being generated by greater deviation. P300 responses are frequently triggered using the “oddball” paradigm or rapid serial visual presentation (RSVP), which involves presenting a series of different stimuli, one of which is infrequent relative to the others (Lees et al., 2018).

The P300 typically occurs about 300 ms after a targeted stimulus and can vary depending on an individual's ability to differentiate events (Farwell and Donchin, 1988; Zhang et al., 2021). The P300 paradigm is regarded as being user-friendly and appropriate for use in BCI applications, particularly those that involve spelling, as it is less likely to cause eye strain (Mendoza-Montoya et al., 2021). Farwell and Donchin introduced the Matrix Speller, which was the first device built on the P300 component (Farwell and Donchin, 1988). Following their work, other studies have suggested diverse paradigms and strong algorithms improve performance, resulting in significant enhancements in both theoretical and experimental approaches (Lotte et al., 2007; Kaufmann et al., 2011; Abiri et al., 2019; Lu et al., 2019, 2020a,b). One of these paradigms is RSVP, which involves presenting a series of stimuli in quick succession, usually at the center of the screen. RSVP can elicit P300 responses when the user sees a target stimulus among distractors (Lin et al., 2018). RSVP has some advantages over the oddball paradigm, such as reducing the number of flashes required to spell a word and avoiding the need for gaze control (Won et al., 2019).

Although the P300 paradigms exhibit promising potential in the BCI field, a significant obstacle is the fact that ERP signals are specific to each subject (Wu et al., 2022). Individuals have varying patterns of brain activity when responding to the same stimuli. As a result, it is necessary to calibrate the system for each person. In the calibration process, the individual is requested to execute a set of tasks that enable the system to acquire knowledge and adjust to their distinctive brain patterns. Although calibration is critical for achieving accurate and reliable results, its time-consuming nature presents a notable challenge for the widespread implementation of BCI.

By utilizing transfer learning (TL) techniques, it is possible to decrease the training time and data needed for a new model, especially when training from scratch is not possible. TL allows machine learning models to be trained on existing datasets from other subjects (known as the source dataset) to apply the acquired knowledge to a new subject (referred to as the target dataset).

One common approach in transfer learning is to adapt a pre-trained model from a source domain to a target domain. Fine-tuning is a variant of this method that utilizes the weights of a pre-trained deep neural network (DNN) based on the source dataset as the initial synaptic configuration for training the target network. Fine-tuning of DNN-based convolutional neural networks (CNNs) is widely used for P300-based BCI applications (Kundu and Ari, 2020a; Kilani et al., 2022), allowing the model to benefit from the general feature extraction capabilities learned from the source dataset. This provides a strong foundation for learning even with limited labeled data from individual subjects and offers faster training times due to the pre-trained weights.

In Kundu and Ari (2020a), a CNN-based TL is introduced to extract high-level features from a fully connected layer of CNN for P300-based character recognition. Then a Fisher-based feature selection technique was employed to achieve the most optimal feature set. Their results demonstrated that a selected set of CNN-extracted deep features outperformed the manually designed features.

Another method in TL is subspace alignment (SA) which aims to increase the distributional similarity between the source and target datasets by aligning the feature distributions between the two datasets (Kundu and Ari, 2020a). Since the source and target datasets have different characteristics in P300 signals, such as variations in the recorded signals, domain adaptation can reduce the need for extensive calibration and training, ultimately resulting in a more efficient and accurate BCI system (Zanini et al., 2018; He and Wu, 2020). Zanini et al. (2018) suggested a method called Riemannian alignment (RA) for aligning EEG data recorded during motor imagery (MI) task. RA calculates the covariance matrices of resting trials and computes their Riemannian mean, and uses it as the reference matrix to align all covariance matrices. To overcome the limitations of RA in terms of flexibility, lower computational cost, and unsupervised nature, He and Wu (2020) proposed a technique called Euclidean alignment (EA) that aligns EEG trials from different subjects in Euclidean space. EA works by identifying a projection matrix that can align the dataset in Euclidean space. The objective is to mitigate the distributional shift between the two domains and enhance the resemblance between their data distributions. In He and Wu (2020) the efficacy of the EA algorithm was assessed on two distinct datasets: one centered on P300 and the other on MI.

In transfer learning, the quality and relevance of source samples utilized for model training can considerably affect the model's performance. To achieve better transfer learning results and avoid negative transfer, it is important to select the most informative and applicable source samples (Wang et al., 2019; Zhuang et al., 2021; Kilani et al., 2023). Selection data from source domains that are more like the target data has also been used in other works. In Wei et al. (2020), the authors adopted a performance-based approach to select the source datasets. A classifier was trained for each source subject, then target data was given to it as evaluation data. Finally, source subjects that exhibited the highest classification performance were selected. While this approach led to improved performance compared to using all source datasets, the challenge of training different classifiers made it difficult to implement in real-time. In Qi et al. (2018), a novel sample selection method using Riemannian geometry measurement has been introduced for P300-based character recognition. A reference epoch was constructed by using a limited number of epochs from target data. The source samples were then selected based on their Riemannian distance to this reference epoch. Their results showed higher character recognition accuracy for sample selection based on Riemannian distance compared to other selection methods. However, Riemannian measurements require more computation, and the choices for classification models are limited compared to Euclidean measurements (He and Wu, 2020; Kilani et al., 2023).

In response to the challenges presented by P300-based BCI, we suggest a new approach to transfer learning that combines the advantages of fine-tuning and subspace alignment. Our approach utilizes finetuning for extracting the high-level features of deep neural networks. Then a domain adaptation technique is used to improve the similarity between the source and target feature distributions. Moreover, to reduce negative transfer, a source sample selection approach is used to choose the samples that are more similar to the target domain data samples. Our transfer learning approach is subsequently implemented on the discriminative restricted Boltzmann machine (DRBM) as the classifier.

Section 2 provides a detailed description of the methods and materials used in our study. We begin by presenting the datasets and explaining the preprocessing steps that were taken to ensure the quality of the data. Next, we describe our proposed approach, which combines the fine-tuning method and subspace alignment using the EA approach. Finally, we outline the process of applying the extracted features using our transfer learning approach to the DRBM classifier. In Section 3, we present the simulation results, including a comparison with other methods and an analysis of the effectiveness of our approach. Finally, in Section 4, we conclude with a summary of our contributions and discuss research in this area.

## 2 Methods and materials

### 2.1 Datasets

We applied the proposed method on two distinct datasets: the *BCI Competition III Dataset II*, derived from a pair of participants, and a *Rapid Serial Visual Presentation (RSVP) Dataset* acquired from *PhysioNet* across 11 participants. A concise summary of each dataset is detailed in Sections 2.1.1 and 2.1.2, respectively. Subsequently, we describe the processing steps and results for each participant.

#### 2.1.1 Dataset 1

**Figure 1:**
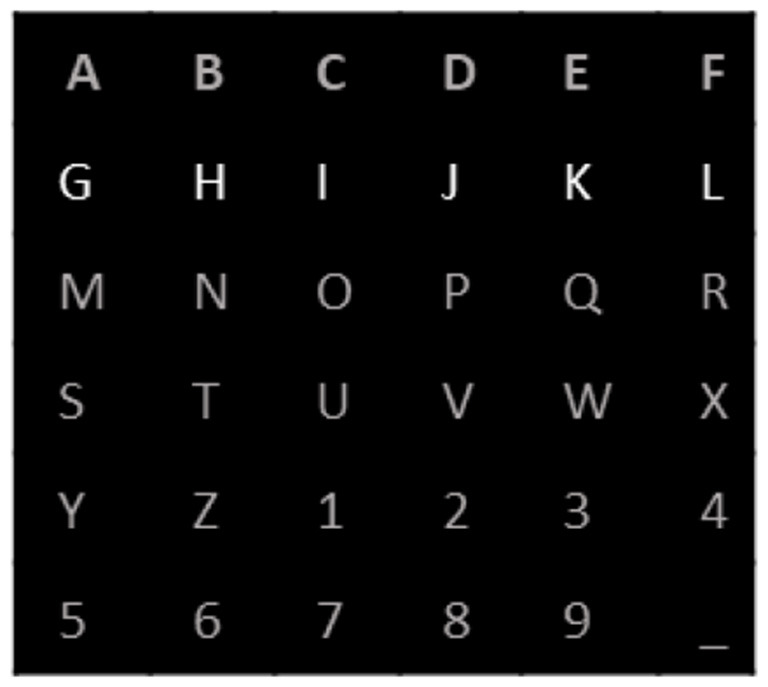
The 6 × 6 alphabet matrix was designed by Farwell and Donchin. All letters and digits are gray and the ones on the intensified rows or columns indicate flashing. In this case, the second row is intensified.

For this study, we employed the BCI competition III dataset II (Blankertz et al., 2006) from the Wadsworth Center, NYS Department of Health. The dataset included EEG data from a pair of participants (A and B), with strict adherence to relevant guidelines and regulations. EEG signals were recorded using 64 scalp electrodes, with a sampling rate of 240 Hz, and a band-pass filter with a bandwidth of 0.1–60 Hz was used to ensure high-quality data. Two individuals who were in good health took part in both training and testing sessions of the P300 speller task experiment. The P300 speller proposed by Farwell and Donchin was used, as shown in Figure 1. Participants were instructed to concentrate on a 6 x 6 matrix speller, which would randomly highlight its rows and columns, resulting in a total of 12 random intensifications. For each character recognition trial in the P300 signal analysis, the 12 intensifications were each repeated 15 times, leading to a total of 180 intensifications. In the EEG signal, the P300 component is generated following each row or column intensification. The objective of the processing was to identify the intersection of P300 component recognition in each row and column of the target character. During each repetition of character recognition, there were two target intensifications and 10 non-target intensifications. During both the training and testing sessions, the participants identified 85 and 100 pre-detected characters, respectively, which were then used for training (from 85 training characters) and testing (from 100 testing characters) the model. During the training session, a total of 2,550 P300 samples (85 characters, each with two target intensifications repeated 15 times) and 12,750 non-P300 samples (85 characters, each with 10 non-target intensifications repeated 15 times) were collected. MATLAB R2019b was used to carry out all analyses.

#### 2.1.2 Dataset 2

Another ERP-based dataset in this study relied on an RSVP dataset acquired from PhysioNet4 (Goldberger et al., 2000). This dataset encompassed EEG recordings from 11 healthy subjects who participated in an experiment involving rapid image presentations at frequencies of 5, 6, and 10 Hz (Matran-Fernandez and Poli, 2017). During the experiment, participants were seated in front of a computer and presented with a series of swiftly changing aerial images of London. These images were categorized as either target images or non-target images. Target images featured a randomly rotated and positioned airplane that had been photo-realistically superimposed, while non-target images lacked airplanes. The main objective was to discern whether the images were target or non-target based on the EEG signals recorded from eight channels, with a sampling rate of 2,048 Hz. Each subject completed two sessions, referred to as “a” and “b,” respectively. In Session “a,” the first image was labeled as “target,” while in Session “b,” it was labeled as “non-target.” For our analysis, we specifically utilized the 5 Hz version (equivalent to five images per second) from Session “a.” The number of EEG samples collected varied across subjects, ranging from 368 to 565, and the target-to-non-target ratio was ~1:9.

### 2.2 Preprocessing

In terms of the preprocessing of the two datasets, we followed the specific recommendations provided in the previously published articles. This ensured that each dataset was prepared in a way that aligned with its unique characteristics and maximized the validity of comparisons between the methods applied. Below we detail the applied preprocessing steps.

To process the EEG signals for Dataset 1, we applied a band-pass filter with a range of 0.1–30 Hz to the signals, as recommended by Thodoroff et al. (2016). Subsequently, we extracted the data by employing a window length of 665 ms following each intensification event. Taking into account the sampling rate of 240 Hz and the number of EEG channels, the input data was then organized into a sequence of samples with dimensions of 160^*^64.

For preprocessing of Dataset 2, the continuous EEG data underwent bandpass filtering between [0.15, 28] Hz, as recommended by Matran-Fernandez and Poli (2017). To streamline processing, we downsampled the EEG signal from the original 2,048 Hz to 64 Hz. Additionally, each trial was epoched to the time interval [0, 0.7] seconds, precisely time-locked to the stimulus onset, to ensure consistent analysis. Here, the data was organized in 8^*^45 images for further analysis as described below.

### 2.3 Deep feature extraction

**Figure 2:**
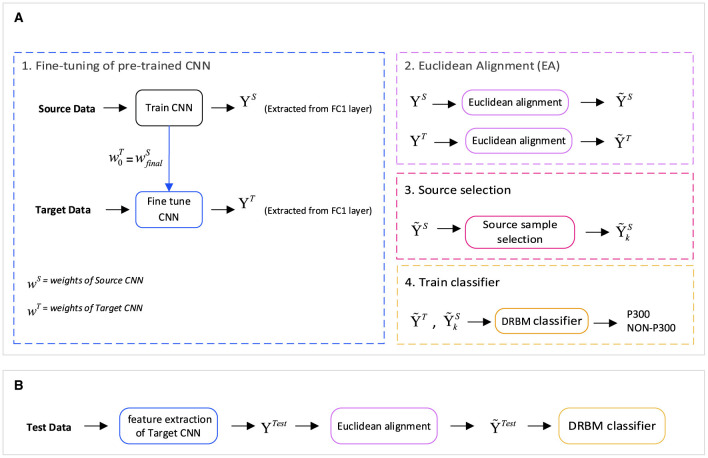
The general structure of the proposed method is based on a transfer learning approach for P300 Signal Classification with Euclidean alignment and fine-tuning of a convolutional neural network. **(A)** The training stage comprises four main modules: fine-tuning of a pre-trained CNN for deep feature extraction, Euclidean alignment to mitigate distribution variations between the source and target domains, source sample selection for optimal representation, and training the discriminative restricted Boltzmann machine (DRBM) classifier using the selected samples. **(B)** During the test stage, features are extracted using the fine-tuning module, followed by transformation into the aligned space, and finally fed into the classification model (DRBM).

We present a new feature extraction method for P300 signal classification. Our approach involves utilizing Euclidean alignment (EA) and a convolutional neural network (CNN) as part of a transfer learning strategy. The proposed method is depicted in Figure 2, which outlines the overall schematic diagram. Initially, the CNN network is trained using the entire source dataset. Subsequently, the weights obtained from the source CNN are used as the initial weights for fine-tuning the CNN with input from the target dataset. Once the network is trained, high-level features are extracted from both CNNs—the source and target feature blocks. Next, we apply the transfer learning-based EA method to align the features in a new Euclidean space, which leads to the creation of similar feature distributions (EA_source and EA_target feature steps). Before assigning the features to the classification, samples from the source that are less similar to the target are removed by the source selection block.

#### 2.3.1 Convolutional neural network

**Figure 3:**
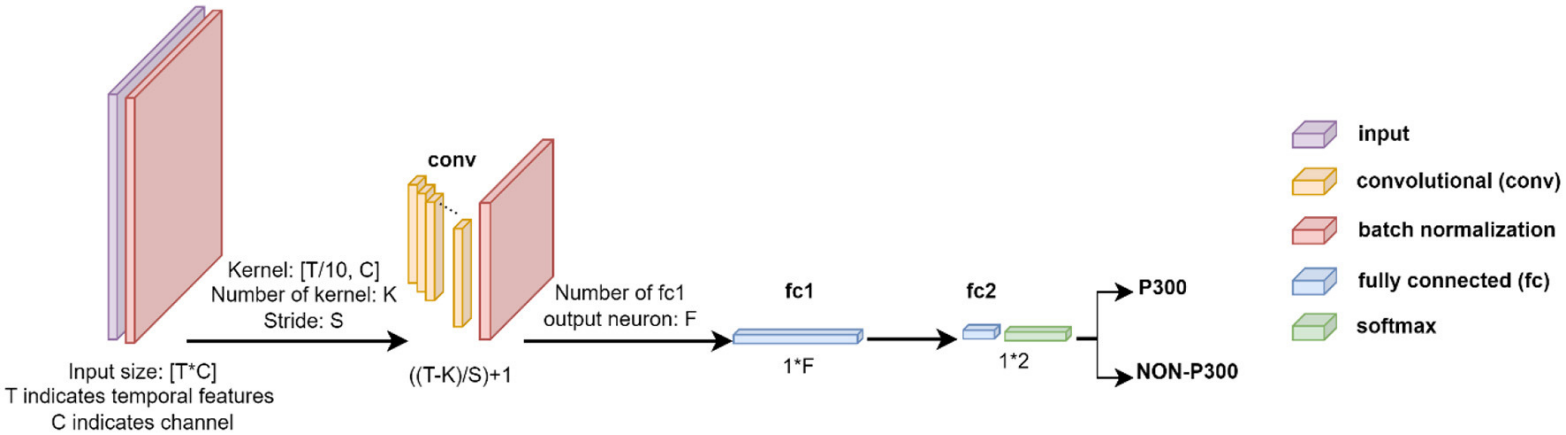
Description of the CNN structure employed for EEG signal classification. TxC indicates temporal features and the number of channels, where T = 160 and C = 64 for Dataset 1, and T = 45 and C = 8 for Dataset 2. The network includes batch normalization in the input layer, followed by a convolutional layer with K kernels of size [T/10xC] and stride of [Sx1] for feature extraction, where K & S = 16 for Dataset 1, and K & S = 5 for Dataset 2. Two Fully Connected layers of f (f = 128 for Dataset 1 and f = 36 for Dataset 2) and two neurons are utilized, followed by a Softmax layer to complete the network. The ReLU activation function is applied throughout the network.

This CNN structure is divided into six layers, including two Batch Normalization (BN) layers, a convolution layer, two fully connected layers, and a Softmax layer as can be seen in Figure 3.

In the first layer, the EEG signal (*X*^*T*×*C*^ where *T* and *C* indicate temporal features and channels, respectively) was fed into the BN layer, which is used to normalize each training mini-batch and accelerate the network training process by reducing internal covariate shifts. After the input data was normalized using the BN layer, the neural network was trained using a convolutional layer. The convolutional layer extracts features using *K* kernels (K = 16 and 5 for Dataset 1 and 2, respectively) and stride of [S × 1] (S = 16 and 5 for BCI competition and RSVP datasets, respectively). Features are extracted by kernels as Equation 1:


(1) $$\begin{array}{l} x^{l}=f{\left(x^{l-1}*w^{l}+b^{l}\right)}^{\;} \end{array}$$

where $x_{\;}^{l-1}$ is the feature of the *l-*1th layer; $w_{\;}^{l}$ and $b_{\;}^{l}$ are filters and biases of the *lth* layer; f*()* is the activation function that introduces a non-linearity aspect to the network. The second BN layer was applied in this step to avoid the convert shifts. Rectified Linear Unit (ReLU) is the most common activation function. Different types of ReLUs can be used. The simple non-linear type of ReLU activates neurons as Equation 2:


(2) $$\begin{array}{l} h\left(x\right)=\max{\left(0,x\right)}^{\;} \end{array}$$

which accepts positive inputs from a neuron and returns 0 for negative input values. Following the extraction of features from the convolutional layer output, the network was completed with two fully connected layers consisting of *f* and 2 neurons, and then a Softmax layer. To assess the model's effectiveness during training and pre-training steps, we utilize the cross-entropy loss function. This function acts as a performance metric throughout the training process by providing necessary gradients for updating weights. Stochastic gradient descent with a learning rate of 0.01 and a momentum factor of 0.9 is used for backpropagation, and L2 regularization parameter of 0.0005 is applied to prevent overfitting.

##### 2.3.1.1 CNN training approach

The paper fine-tuned a pre-trained CNN on a new task, adjusting its weights for better adaptation while retaining prior knowledge. This enabled quick and accurate adaptation to new tasks with limited data, demonstrating CNN's ability to learn versatile features during pre-training. In this study, two subjects were considered for the Dataset 1, with one as the source and the other as the target. It consisted of 15,300 training samples (85^*^12^*^15), and all samples from the source dataset were used for the initial training (pre-training) of the CNN according to the aforementioned structure. Then, initial convolutional layers of the pre-trained CNN were frozen to retain their generic feature extraction capabilities learned from the source data. These frozen layers were then used to initialize the corresponding layers in the target (current) CNN. In the next step, only 7,200 samples (40^*^12^*^15) from the target dataset were used to retrain the model. This approach allowed for leveraging the pre-trained network's knowledge to adapt it to the target task, thereby improving the accuracy and reducing the calibration time (a reduction of 53%).

In Dataset 2 (RSVP), which comprises 11 subjects, our primary objective is to select the most suitable source subject for each target subject. To accomplish this, we employed a source subject selection process based on performance evaluation. The approach involved training 10 classifiers, each using the training dataset from one of the 10 source subjects. These classifiers were subsequently tested using 20% of the training data from the target subject. After conducting the tests, we identified the source subject whose classifier achieved the highest classification accuracy out of the 10 groups. This source subject was then chosen as the optimal match for the respective target subject. By employing this methodology, we aimed to ensure that the most competent and relevant sources were utilized to enhance the overall performance of the system. Notably, 10% of training selected source datasets were used for tuning the parameters as a cross-validation step in Dataset 1 and 2 analyses.

#### 2.3.2 Euclidean space data alignment

According to the literature (He and Wu, 2020), an unsupervised approach was proposed to align EEG data of various subjects in Euclidean space with a focus on enhancing their similarity which is called Euclidean alignment (EA). In this study, we applied this EA approach to the features extracted from the first fully connected layer of the CNN structure [y = f(X)] in both the source and target datasets to improve the efficiency of the transfer learning process. The EA technique computed the reference matrix based on the covariance of a subject's *N*-trial feature, as given by Equation 3:


(3) $$\begin{array}{l} \bar{R}=\frac{1}{N}\overset{N}{\underset{n=1}{\sum }}{\;y}^{n}{\left(y^{n}\right)}^{T} \end{array}$$

After computing the reference matrix for each dataset, the datasets were aligned using the Equation 4:


(4) $$\begin{array}{l} {ỹ}^{n}={\bar{R}}^{-\frac{1}{2}}{\;y}^{n} \end{array}$$

This method is computationally efficient, does not require labeling, and results in distributions of the aligned data being more similar. To implement the EA, we calculated the reference covariance matrix in two ways offline and online calculation. In offline calculation, all unlabeled trials from a new subject are available and used to calculate the reference, while in online calculation only one trial of unlabeled data at each step is used to update the reference covariance matrix.

#### 2.3.3 Source sample selection

To reduce the negative effect of transfer from the source domain to the target domain, source sample selection has been used in the current study. This process involves selecting the most relevant and informative source samples to improve the performance of the target domain. There are different criteria for choosing source data. Here we employed an unsupervised sample selection approach based on distance criteria. The distance-based method involved creating a reference for the target subject by averaging its feature samples. The Euclidean distance measurement was used to calculate the distance between each source feature sample and the reference. The samples with the smallest distance were then selected. Algorithm 1 provides a more detailed description of the introduced source selection method.

**Algorithm 1:** Source sample selection.

**Input:** • ${ỹ}_{j}^{T}$ is the extracted feature vector from j*th* target sample. • ${ỹ}_{i}^{S}$ is the extracted feature vector from i*th* source sample. • *N*_*T*_ is the number of target samples. • *N*_*S*_ is the number of source samples. • *k* is the number of source samples to be selected
Calculate target reference: $\text{r}=\frac{1}{N_{T}}\sum_{j=1}^{N_{T}}{ỹ}_{j}^{T}$; **for** *i* = l: *N*_s_ **do** $\text{d}\left(j\right)=\left(\text{r}-{\tilde{\text{y}}}_{\text{i}}^{\text{S}}\right){\left(\text{r}-{\tilde{\text{y}}}_{\text{i}}^{\text{S}}\right)}^{{\;}^{\prime }};$ **end** Sort **d** and select *k* source samples with the smallest distance.
**Output:** *k* index from source (***Ỹ*^*S*^**)

### 2.4 Classification

Restricted Boltzmann Machines (RBMs) are an energy-based model with hidden variables. RBMs are typically used as generative models, meaning they can learn to generate new samples from a given distribution. The model learns to assign higher probabilities to samples that are similar to the ones it has seen during training and lower probabilities to dissimilar samples.

For classification problems, a Discriminative RBM (DRBM) was proposed (Larochelle and Bengio, 2008). Unlike a standard RBM, a DRBM is trained to directly model the conditional distribution of the labels given the input data. This makes the DRBM a discriminative model, as it is trained to predict the labels of new samples, rather than generate them. DRBMs have been shown to achieve state-of-the-art performance on several benchmark datasets.

Previous studies have reported the successful performance of a hybrid form of DRBM in the P300 classification (Varsamou and Antonakopoulos, 2019; Kordmahale et al., 2022; Aghili et al., 2023). Therefore, we have adopted this approach, using 10 hidden neurons, to classify features extracted from the output of the convolutional layer.

## 3 Results and discussion

We utilized two datasets (dataset II from BCI competition III and RSVP dataset) to evaluate the proposed method, as introduced in Section 2.1. In the initial phase, we showcase our effectiveness using Dataset 1, illustrating our commendable performance in both offline and online scenarios (more details about the scenarios in Section 2.3.2). Subsequently, in the second phase, we present the findings obtained from Dataset 2, providing more comprehensive insights through ablation experiments. Given the larger pool of subjects in this dataset, we can effectively showcase our robustness in this context.

### 3.1 Dataset 1 results

#### 3.1.1 Data visualization

**Figure 4:**
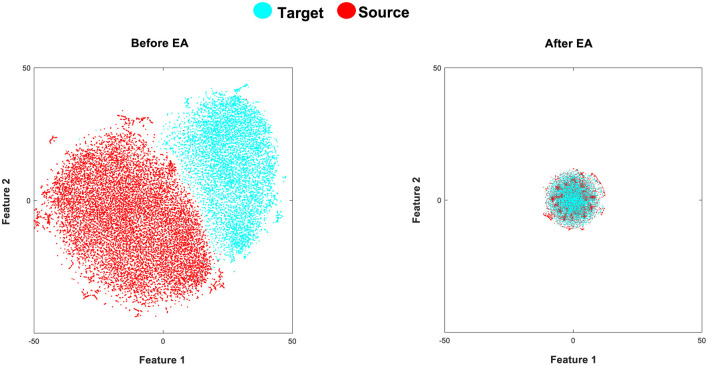
**(Left)** presents a t-SNE visualization of the data distributions in the source (red color) and target (cyan color) domains within Dataset 1. The target involves data samples of 40 characters from subject A, and the source distribution comprises data samples of 85 characters from subject B, after extracting fine-tuned CNN features (before applying EA). On the other hand, the **(Right)** depicts the target and source feature distributions after applying EA (fine-tuned CNN+EA).

The t-Stochastic Neighbor Embedding (t-SNE) is a visualization technique that maps high-dimensional data to a two or three-dimensional (2D or 3D) space (Van Der Maaten and Hinton, 2008). The goal of t-SNE is to optimize the pairwise distances in the reduced space for the distances in the original manifold. In our case, we aim to represent each extracted feature in a 2D space to better appreciate the effect of the EA alignment in the cross-subject shifts. In Figure 4, we aim to illustrate the extracted features by the fine-tuned CNN before and after applying EA to demonstrate their impact on the similarity of target and source distributions. As shown in Figure 4, the proposed TL (fine-tuned CNN+EA) method demonstrates a suitable effect on the similarity of target and source distributions.

#### 3.1.2 Performance evaluation

**Figure 5:**
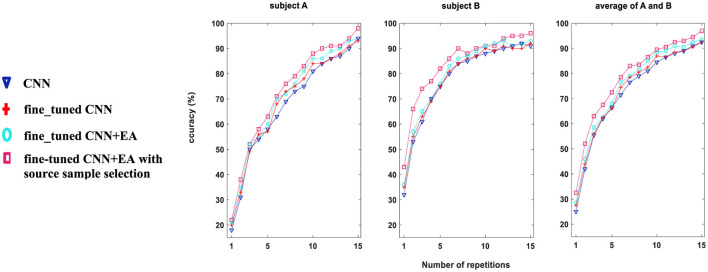
Comparative analysis of character recognition accuracy (%) for Dataset 1, subjects A and B, using three distinct methodologies for training on 40 target characters. Approach (i) employs a conventional CNN (blue); approach (ii) applies the fine_tuned CNN method (red); approach (iii) utilizes the fine_tuned CNN+EA method without source sample selection (cyan); and approach (iv) applies the fine-tuned CNN+EA with source sample selection (pink color).

In Figure 5, the character recognition performance was evaluated on Dataset 1, subjects “A” and “B” using three different feature extraction approaches as comparison study (DRBM was set as a classification method for all of them): (i) conventional CNN technique without fine-tune approach with 40 target characters, (ii) fine-tuning approach is used for 40 target characters training, (iii) fine_tuned CNN+EA without sources samples selection, and (iv) proposed TL approach (7,200 sources samples were selected by Algorithm 1). The results show that the accuracy of character recognition is higher with the proposed approach compared to fine-tuned CNN+EA, fine-tuned CNN, and conventional CNN. By selectively utilizing 7,200 source samples by the proposed sample selection method, we not only boost the system's accuracy but also enhance the efficiency of the training process by ensuring that only the most relevant and impactful data is utilized. The results underscore the importance of source sample selection in improving the efficacy of TL techniques in BCI applications.

To evaluate the performance of the proposed algorithm, we compared its results with those of previous works that reported their findings on the benchmark dataset (BCI competition III dataset II), which included the CNN-1 (Cecotti and Gräser, 2011), MCNN-3 (Cecotti and Gräser, 2011), MsCNN-TL-ESVM (Kundu and Ari, 2020b), and DRBM (Larochelle and Bengio, 2008) techniques. Table 1 summarizes the results of the current character recognition methods compared to our proposed method. Our approach achieved excellent classification performance, with an accuracy of 98%, and 96% for subjects A and B after 15 repetitions, respectively. It's worth noting that these results were achieved using only 40 training characters to train the model.

**Table 1:** The accuracy of character recognition (%) is compared between our proposed method and other existing methods for subjects A and B from Dataset 1.

**Subjects**	**Methods**	**Repetitions**														
		**1**	**2**	**3**	**4**	**5**	**6**	**7**	**8**	**9**	**10**	**11**	**12**	**13**	**14**	**15**
A	^*^ **Proposed method**	22	**38**	**52**	**58**	**63**	**71**	76	**79**	83	**88**	90	**91**	91	**94**	**98**
	CNN-1	16	33	47	52	61	65	77	78	**85**	86	90	**91**	91	93	97
	MCNN-3	17	35	50	55	63	67	**78**	**79**	84	85	**91**	90	**92**	**94**	97
	^*^MsCNN-TL-ESVM	**24**	**38**	46	50	60	70	72	**79**	84	86	89	89	**92**	**94**	96
	^*^MsCNN-ESVM	16	16	39	38	46	49	65	69	78	81	82	87	88	89	89
	DRBM-85	19	35	47	57	62	65	74	78	83	85	86	89	91	92	94
	^*^DRBM-45	20	31	43	50	55	61	70	72	78	82	80	84	85	89	91
B	^*^ **Proposed method**	**43**	**66**	**74**	**77**	**82**	**86**	**90**	88	90	91	91	94	95	95	**96**
	CNN-1	35	52	59	68	79	81	82	89	92	91	91	90	91	92	92
	MCNN-3	34	56	60	68	74	80	82	89	90	90	91	88	90	91	92
	^*^MsCNN-TL-ESVM	40	59	67	74	79	84	**90**	**92**	**94**	**97**	**96**	**98**	**97**	**97**	**96**
	^*^MsCNN-ESVM	37	58	65	73	74	83	87	88	91	94	92	92	93	96	**96**
	DRBM-85	32	50	62	65	75	77	83	86	90	93	94	95	95	94	93
	^*^DRBM-45	32	45	52	65	73	76	82	84	86	92	92	93	93	91	89

^*^Indicates that 40 target characters (7,200 training target samples) are used for training the model. Other methods utilized all 85 target characters (15,300 training target samples) for training the model.

The best accuracy for each repetition was made bold for clarification.

**Figure 6:**
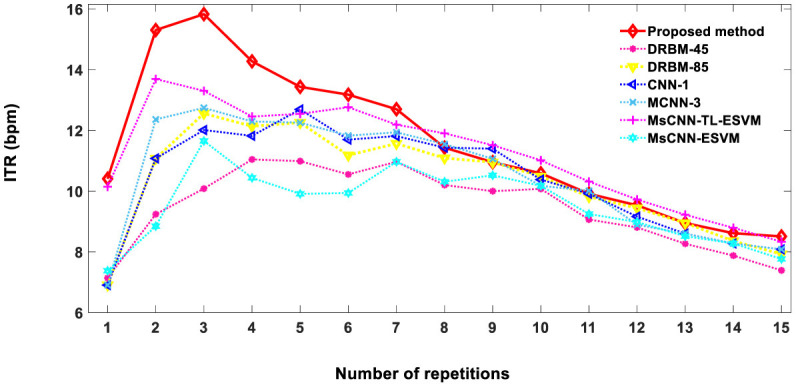
The comparison of information transfer rates (ITRs) between the proposed method and other techniques, based on the average accuracy of two subjects A and B from Dataset 1. DRBM-45 and DRBM-85 indicate that 45 and 85 training characters are used for training the model, respectively.

The speed and accuracy of a user's communication with a computer using brain signals can be quantified using the information transfer rate (ITR) formula. ITR is measured in bits per minute (bpm) and is defined as Equation 5.


(5) $$\begin{array}{l} ITR=\frac{\;\left(\left(\log_{2}N+P\log_{2}P+\left(1-P\right)\log_{2}\frac{1-P}{N-1}\right)\times 60\right)}{T} \end{array}$$

where N is the number of characters in the BCI paradigm, which is 36 in this case. P is the character recognition accuracy, and T is the time required for character recognition, defined as Equation 6:


(6) $$\begin{array}{l} T=2.5+\left(\left(0.100\;s\;+\;0.075\;s\right)\times 12\right)\times Nr\;,\;1\leq Nr\;\leq 15 \end{array}$$

where Nr is the number of repetitions (1 ≤ Nr ≤ 15). Figure 6 demonstrates the ITR of the proposed method in comparison to other previously reported methods. ITR values of 10.4, 13.4, 10.6, and 8.5 bpm were achieved for 1, 5, 10, and 15 repetitions, respectively. The proposed technique achieves an optimal level of character recognition and speed for 1–7 repetitions, resulting in a higher ITR score than other methods. The practicality of our approach extends to real-time applications, offering a robust solution to the prevalent speed limitations in BCI systems.

#### 3.1.3 Online vs. offline analysis

The difference between online and offline computations lies in the covariance matrix calculation, as explained previously (Section 2.3.2). The research study highlights the outcomes of online character recognition in two subjects, identified as Subject A and Subject B. These results are comprehensively summarized in Table 2, presenting an overview of the achieved recognition accuracy in real-time scenarios. By serving as a valuable reference, Table 2 provides crucial insights into the effectiveness of online character recognition among different individuals. The implications drawn from these findings hold significant potential for advancing the field of online character recognition and its practical applications.

**Table 2:** The accuracy of character recognition (%) using online and offline methods is evaluated for subjects A and B from Dataset 1.

**Subjects**	**Analysis approach**	**Repetitions**														
		**1**	**2**	**3**	**4**	**5**	**6**	**7**	**8**	**9**	**10**	**11**	**12**	**13**	**14**	**15**
A	Offline	22	38	52	58	63	71	76	79	83	88	90	91	91	94	98
	Online	23	37	51	62	63	72	74	79	84	86	87	91	92	93	97
B	Offline	43	66	74	77	82	86	90	88	90	91	91	94	95	95	96
	Online	39	64	72	77	82	87	89	87	89	91	90	95	95	97	97

Experimental results show that the *t*-test conducted between the offline and online results (*P*-value = 0.25) yielded non-significant differences. This outcome provides strong evidence that the proposed method is highly suitable for online implementation. Moreover, the system's efficiency is further highlighted by the reduced number of repetitions required to achieve satisfactory results. These smaller number of repetitions demonstrate the speediness of the system, making it an efficient and practical solution for P300 signal classification tasks.

### 3.2 RSVP results

Given the significant class imbalance in the RSVP dataset, we opted to employ balanced classification accuracy (BCA) as the performance metric, as recommended in the literature (He and Wu, 2020). To elaborate, let's designate “m1” as the true number of trials from the target class and “m2” as the true number of trials from the non-target class. Additionally, “n1” and “n2” represent the number of trials correctly classified as target and non-target, respectively, by the algorithm. To compute the BCA, we follow these steps:


$$\begin{array}{l} a1=\frac{n1}{m1}\;a2=\frac{n2}{m2} \end{array}$$

In this context, “a1” refers to the classification accuracy of the target class, while “a2” represents the classification accuracy of the non-target class.


$$\begin{array}{l} BCA=\frac{a1+a2}{2} \end{array}$$

**Figure 7:**
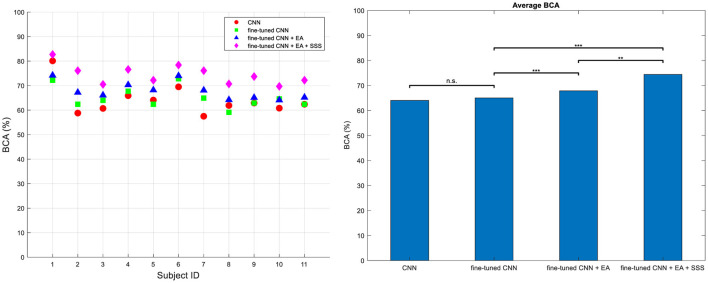
BCA values for all subjects **(left)** and their average **(right)** across four distinct training methodologies in Dataset 2. Approach (i) utilizes a conventional technique without fine-tuning, training on target characters (CNN); approach (ii) involves a fine-tuned CNN for feature extraction (Finetune-CNN); approach (iii) employs the proposed TL method without source sample selection (Finetune-CNN+EA); and approach (iv) incorporates the fined-tune CNN+EA method with source sample selection (Finetune-CNN+EA+SSS). Significance levels are denoted by *** for *P* < 10^−3^ and ** for *P* < 10^−2^.

Figure 7 presents the BCA of the methods across four different types of analysis for Dataset 2, highlighting the improvements made in our final approach. The classifier is DRBM for all feature extraction methods. The first category termed conventional CNN without a fine-tuning approach to the target subjects' dataset. In the fine-tuned CNN approach, we aimed to show the effect of fine-tuning on the performance by employing a fine-tuned CNN, utilizing data from source subject, to extract optimal features from the target subject' dataset. The third category demonstrates the use of fine-tuned CNN combined with EA without employing the source selection strategy. Here, we aim to highlight the impact of EA. The features derived from both the source CNN and the target fine-tuned CNN were transformed into the Euclidean space using EA. Subsequently, the features transferred from the target, along with all the transferred features from the source, were inputted into the classifier. In the fourth approach, we present our proposed method, which integrates fine-tuned CNN with EA along with the proposed source selection approach. Ultimately, our method yields significantly higher BCA (Balanced Classification Accuracy) values compared to the other three methods. These results were evaluated using the one-way ANOVA statistical test followed by Tuckey's hsd *post-hoc*, confirming the superiority of our proposed approach (*p* < 0.001).

**Figure 8:**
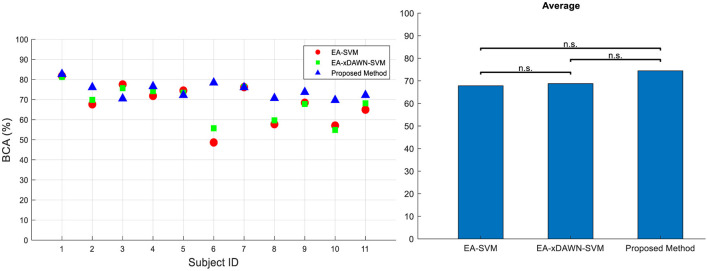
BCA values for all subjects **(left)** and their average **(right)** for three different approaches in Dataset 2: (i) EA-SVM, (ii) EA-xDAWN-SVM, and (iii) proposed method.

We further conducted a comprehensive comparison of our newly developed method with two existing approaches using the RSVP dataset, as reported in the literature (He and Wu, 2020). We also followed the same evaluation criteria and procedures outlined in He and Wu (2020) to make the results directly comparable. The two approaches we assessed included EA-SVM and EA-xDAWN-SVM. For each method, we measured its accuracy and effectiveness in analyzing EEG data on the RSVP dataset. This comparison is represented in Figure 8. These findings provide strong evidence for the potential superiority of our method in analyzing EEG data from the RSVP dataset. Specifically, we achieved a remarkable increase of 5.7 and 6.65%, respectively, in BCA values compared to EA-xDAWN-SVM and EA-SVM, which were the best-performing existing approaches.

## 4 Conclusion

This study introduced a new transfer learning approach for the ERP-based brain-computer interface that incorporates source sample selection to improve the performance of the system further. Source sample selection in transfer learning has several advantages. It can help to reduce the negative effects of domain shift, where there are differences between the distribution of data in the source and target domains. By selecting appropriate sources, it is possible to mitigate these negative effects and improve performance on the target task. The proposed selection approach chooses the most relevant source samples based on their similarity to the target samples to enhance the transferability of the model further.

In addition, the proposed approach utilized fine-tuning and data alignment techniques to improve the performance of the P300 BCI, especially when dealing with limited labeled data. Specifically, the approach fine-tunes a pre-trained model on a similar task using a small amount of labeled data and aligns the data distributions between the source and target domains to minimize the domain shift. The results of our experiments showed that the proposed approach outperforms the baseline models and achieves state-of-the-art performance on the datasets used in this study.

Furthermore, the proposed approach is shown to be generalizable to other datasets, demonstrating its potential for wider applicability. One of the most important aspects of the proposed method is extracting high-level features in a new domain (EA) while minimizing the difference between both feature groups of source and target. Importantly, while Euclidean Alignment has been explored in the context of transfer learning for BCIs, our work demonstrates its novel application to high-level features extracted from convolutional neural networks. This approach directly addresses the inherent variability of EEG signals between subjects, facilitating more robust knowledge transfer and improving performance compared to methods that focus solely on raw time samples. The findings of this study suggest that fine-tuning, data alignment, and source sample selection could be promising techniques for enhancing the performance of ERP-based BCIs while reducing calibration time and could pave the way for further research in this area.

## Data availability statement

The original contributions presented in the study are included in the article/supplementary material, further inquiries can be directed to the corresponding author.

## Ethics statement

Ethical approval was not required for the study involving humans in accordance with the local legislation and institutional requirements. Written informed consent to participate in this study was not required from the participants or the participants' legal guardians/next of kin in accordance with the national legislation and the institutional requirements.

## Author contributions

SK: Conceptualization, Formal analysis, Investigation, Methodology, Software, Validation, Visualization, Writing – original draft, Writing – review & editing. SA: Conceptualization, Formal analysis, Investigation, Methodology, Software, Supervision, Writing – original draft, Writing – review & editing. YF: Supervision, Writing – original draft, Writing – review & editing. AS: Supervision, Writing – original draft, Writing – review & editing.
